# Supplementary material for: Unraveling Signatures of Local Adaptation among Indigenous Groups from Mexico
Source: Genes (Basel). 2022 Nov 30;13(12):2251. doi: 10.3390/genes13122251 (PMC9778281; doi:10.3390/genes13122251)
Supplement: Supplementary file 1 [file genes-13-02251-s001.zip › Supp_Fig_Garcia_Ortiz_2022.pdf]

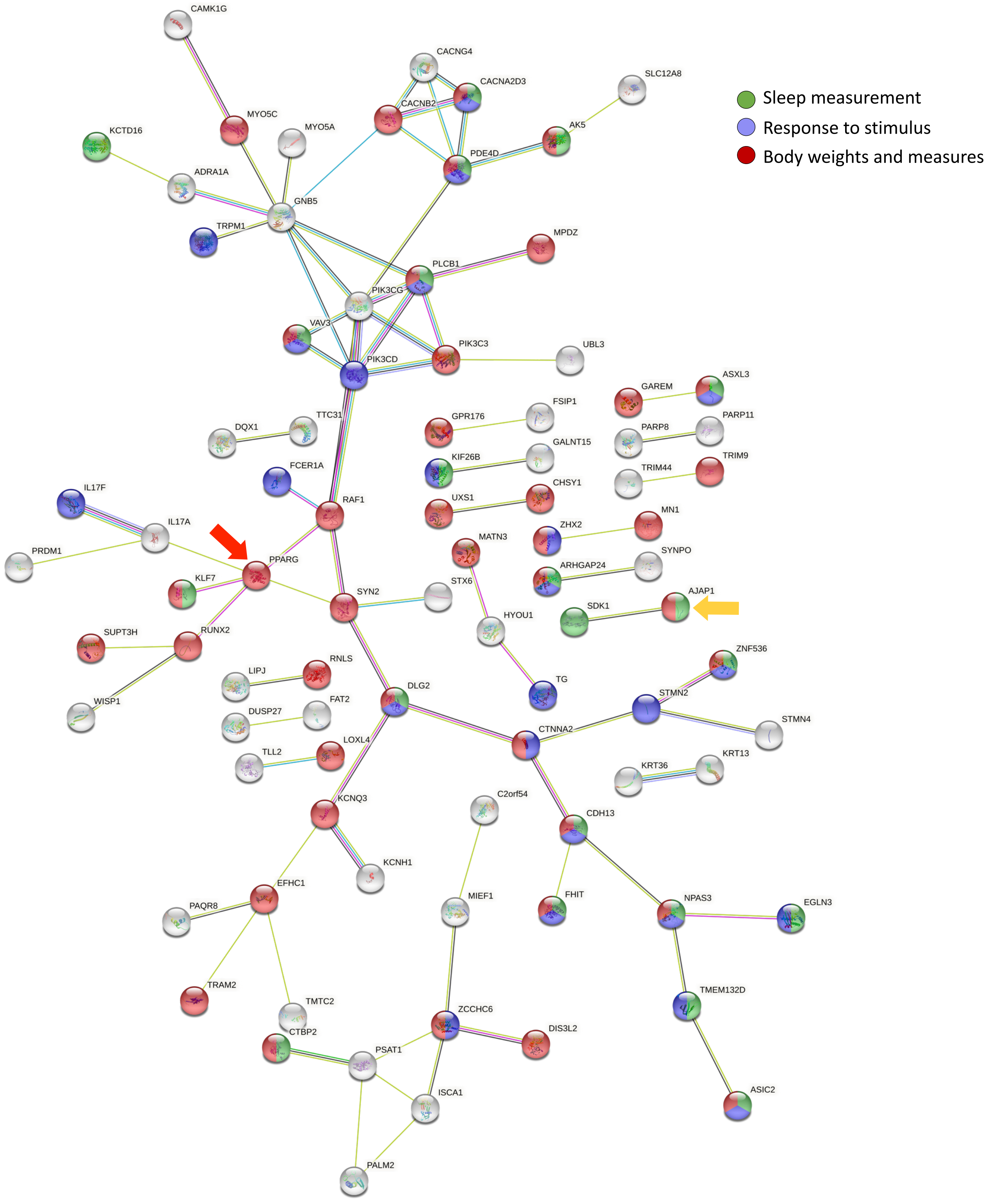


Figure S1. PPI network for genes under selection identified in the North.

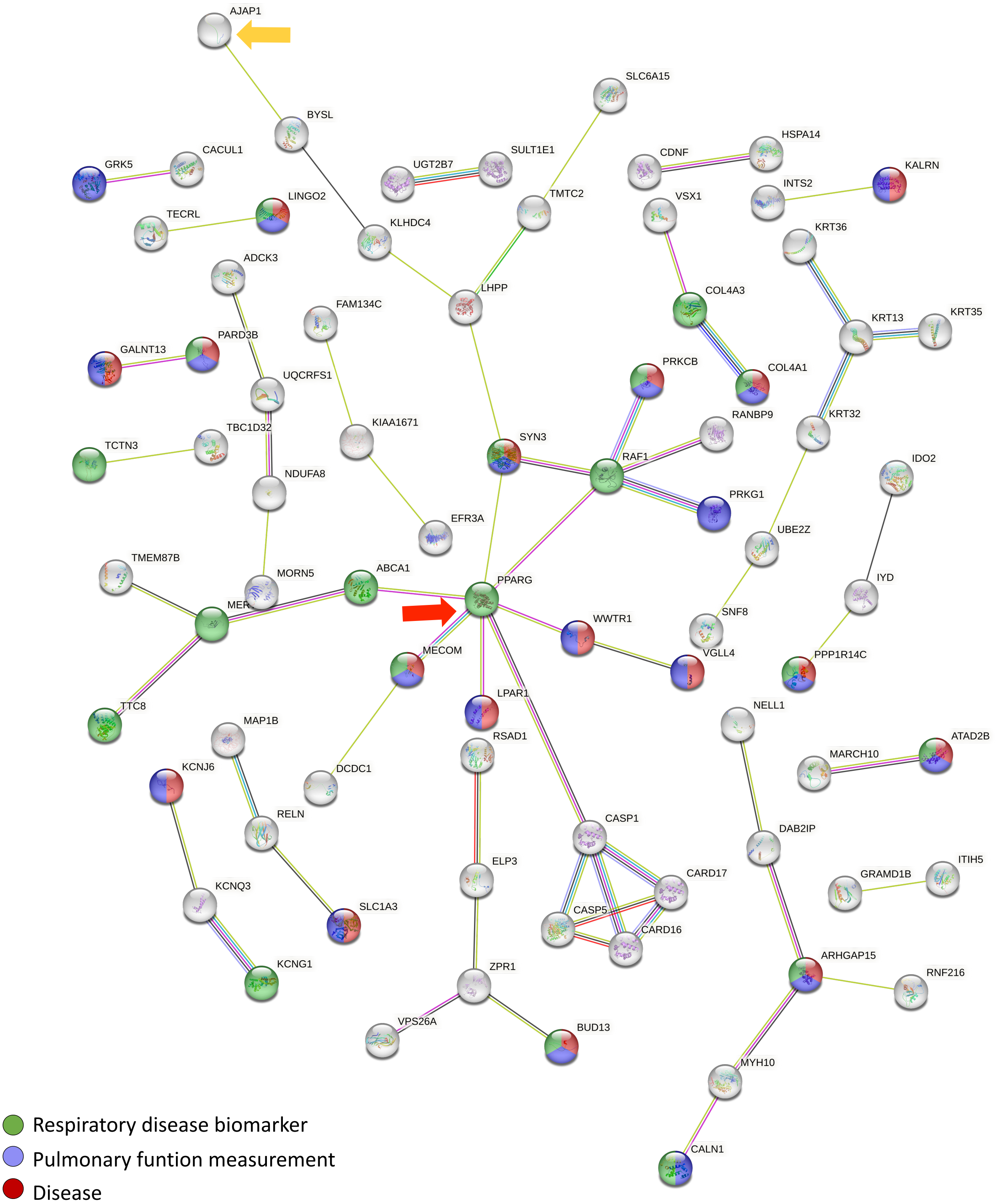


Figure S2. PPI network for genes under selection identified in the Northwest.

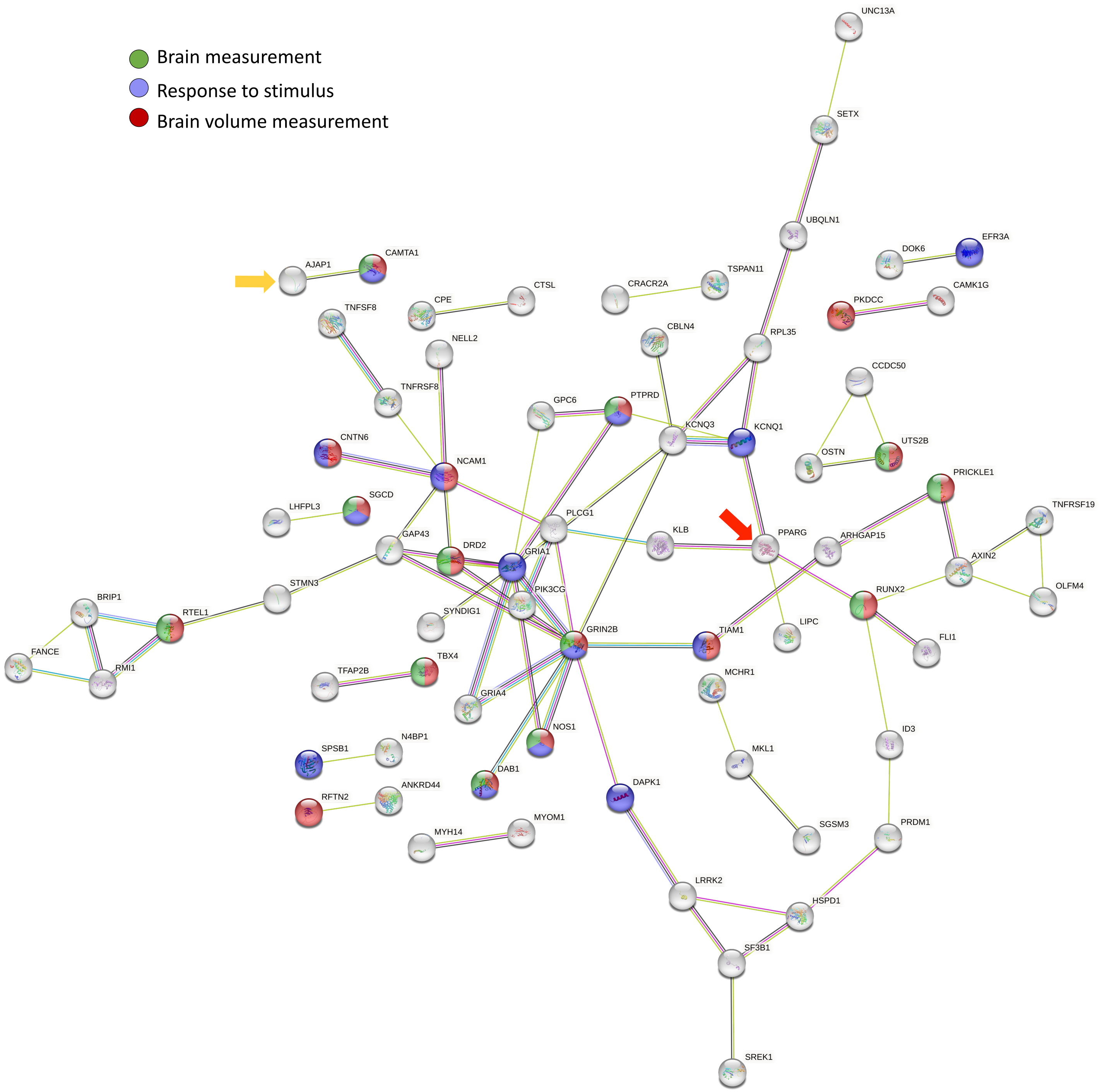


Figure S3. PPI network for genes under selection identified in the Center.

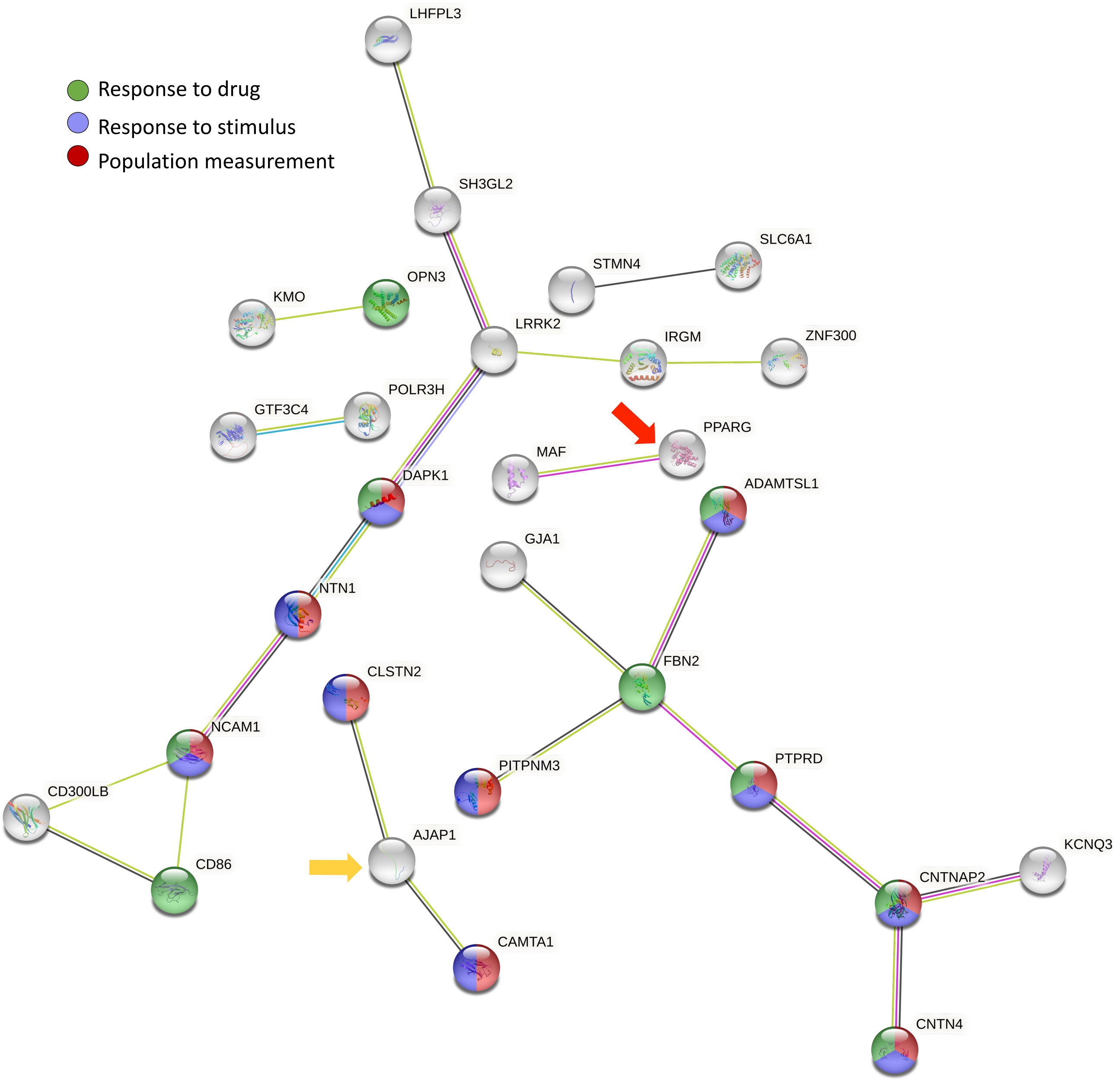


Figure S4. PPI network for genes under selection identified in the South.

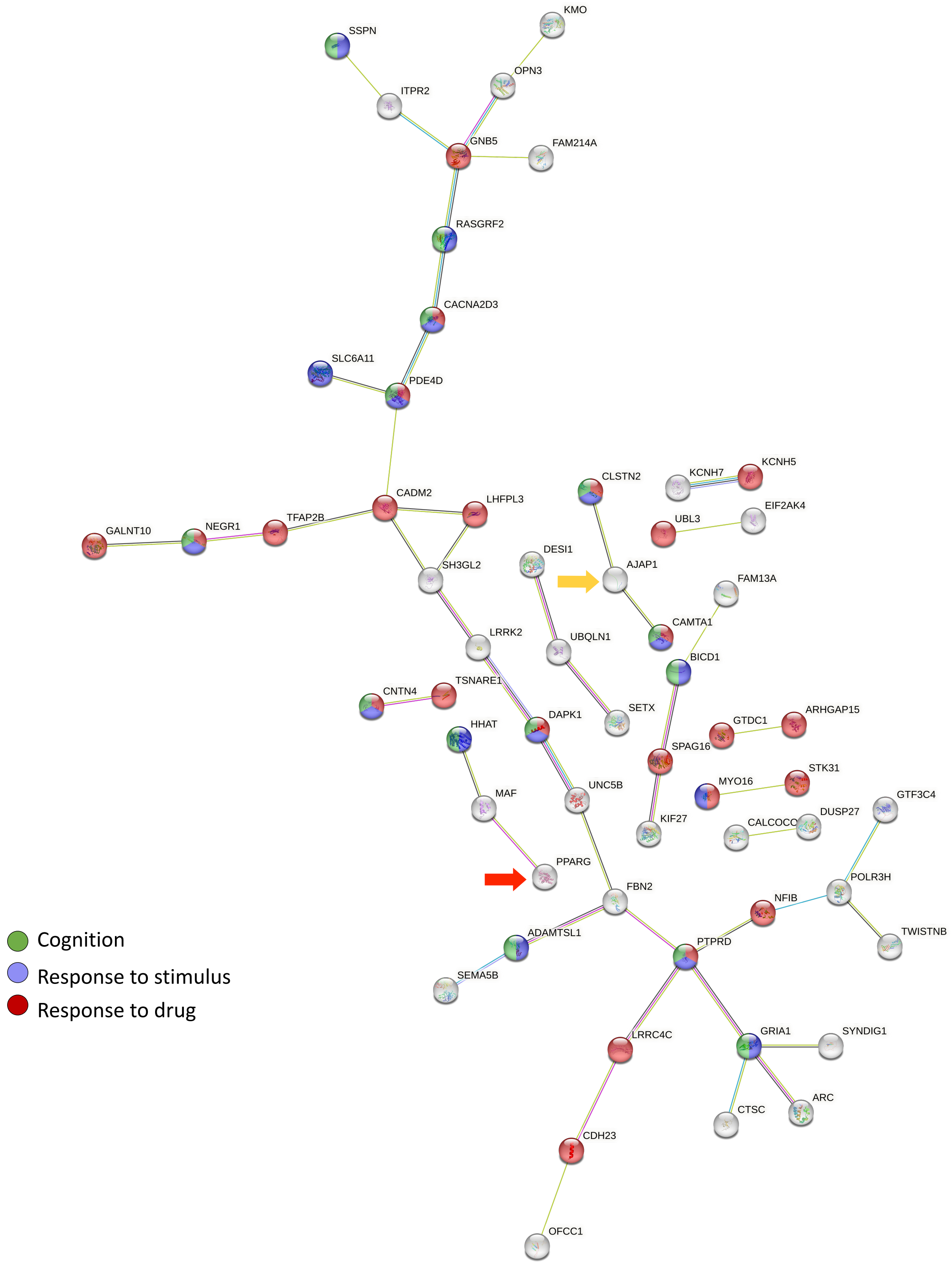


Figure S5. PPI network for genes under selection identified in the Southeast.
